# Silencing PPA1 inhibits human epithelial ovarian cancer metastasis by suppressing the Wnt/β-catenin signaling pathway

**DOI:** 10.18632/oncotarget.19346

**Published:** 2017-07-18

**Authors:** Haiying Niu, Wei Zhou, Yingxi Xu, Zhiqi Yin, Wenzhi Shen, Zhen Ye, Yanhua Liu, Yanan Chen, Shuang Yang, Rong Xiang, Lina Wang, Pengpeng Qu

**Affiliations:** ^1^ Department of Gynecology and Obstetrics, Tianjin First Center Hospital, Tianjin, China; ^2^ Department of Immunology, Nankai University School of Medicine, Tianjin, China; ^3^ State Key Laboratory of Experimental Hematology, Institute of Hematology and Blood Disease Hospital, Chinese Academy of Medical Sciences, Tianjin, China; ^4^ Department of Pathology, Tianjin First Center Hospital, Tianjin, China; ^5^ Department of Gynecology Oncology, Tianjin Central Hospital of Gynecology Obstetrics, Tianjin, China

**Keywords:** PPA1, epithelial ovarian cancer, metastasis, Wnt/β-catenin

## Abstract

Inorganic pyrophosphatase (PPA1) activity is a key determinant of cellular inorganic pyrophosphate levels, and its expression is correlated with growth of several solid tumors. To investigate this relationship, we first examined PPA1 expression in human epithelial ovarian cancer (EOC) samples, and found that PPA1 was overexpressed in tumors from EOC patients. Higher PPA1 levels correlated with advanced grades, stages, and poor survival in EOC patients. Examination of PPA1 function in EOC revealed that silencing PPA1 inhibited EOC migration, epithelial-mesenchymal transition (EMT), and metastasis *in vitro* and *in vivo*. In addition, PPA1 may promote the dephosphorylation and translocation of β-catenin. These results demonstrate that silencing PPA1 inhibits EOC metastasis by suppressing the Wnt/β-catenin signaling pathway. Strategies for downregulating PPA1 may have therapeutic potential for the prevention and treatment of EOC.

## INTRODUCTION

Epithelial ovarian cancer (EOC) accounts for more than 90% of ovarian cancers. Despite continued efforts to improve EOC treatment, it is still the fifth leading cause of cancer death among women [1]. The high mortality of EOC patients is mainly attributed to metastasizing to the abdominal viscera. Metastasis is a multistep process that includes epithelial–mesenchymal transition (EMT) of tumor cells, collective invasion, reorganization of cytoskeleton, and microenvironment remodeling [2, 3]. Better understanding of the molecular mechanisms involved in EMT is important for identifying novel therapeutic targets and establishing new treatment strategies for EOC.

Inorganic pyrophosphatase (PPA1) has been identified as a soluble cytosolic pyrophosphatase, which was found to be essential for growth and development in the round-worms Ascaris and Caenorhabditis elegans [4, 5]. Among mammals, PPA1 has the potential to regulate neurite growth via JNK dephosphorylation in mouse neuroblastoma cells [6], as well as the potential to induce type I collagen synthesis and stimulate calcification by osteoblasts [7]. Increases in the expression and activity of PPA1 in rat and mouse livers are correlated with aging [8, 9].

PPA1 has been detected in all tissues examined with Northern blots, which indicates that PPA1 acts largely as a housekeeping gene [10]. PPA1 was upregulated in neoplasms such as colorectal cancer [11], lung adenocarcinoma [12], prostate cancer [13], hepatocellular carcinoma [14], breast cancer [15], and ovarian cancer [16]. Sang et al. found PPA1 overexpression correlated with tumor progression and poor survival of patients. They also found that PPA1 could inhibit the migration and invasion of gastric cancer cells *in vitro*. Their findings indicate that PPA1 might be a useful marker for gastric cancer metastasis and progression [17]. In previous studies, we performed a proteomic analysis in ovarian cancer tissues to identify proteins that were differentially expressed in ovarian cancer tissues compared to normal ovarian tissues. We identified PPA1 as a potential EOC marker [18].

We hypothesized that PPA1 is correlated with metastasis and a poor EOC prognosis. In this study, PPA1 expression levels were evaluated in EOC tissues from 55 EOC patients and functional experiments were performed to confirm the metastatic features of PPA1. Finally, the molecular role of PPA1 in regulating Wnt/β-catenin-associated EMT signaling was explored.

## RESULTS

### The expression of PPA1 in EOC patients

**Figure 1:**
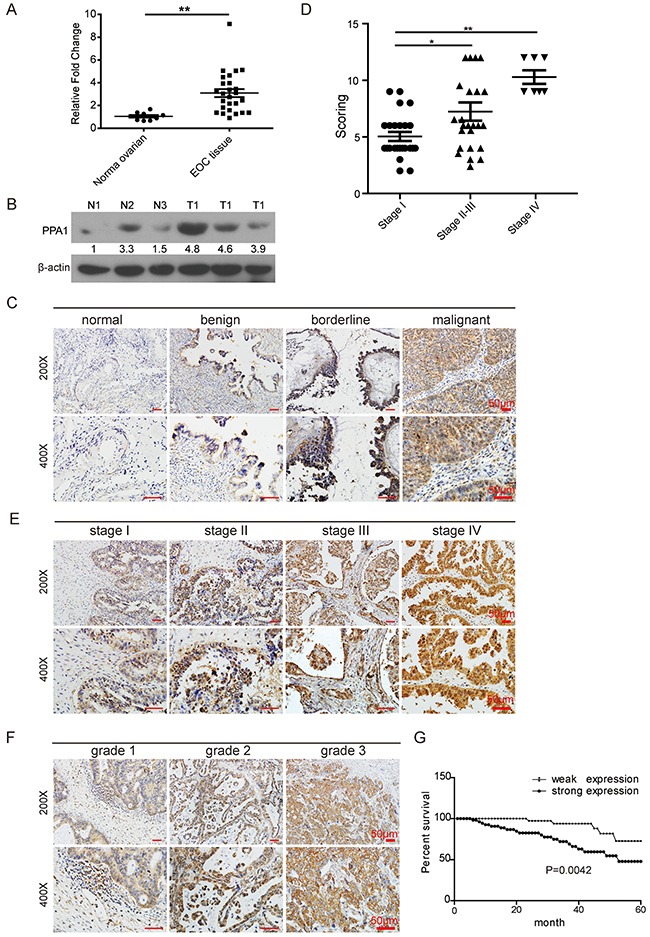
Validation of PPA1 expression in EOC **(A)** Expression of PPA1 mRNA in fresh EOC tissues and normal ovarian tissues tested with qRT-PCR. **(B)** Expression of PPA1 validated by western blot analysis in three individual EOC tumor samples (T: tumor samples) and three individual normal samples (N: normal ovarian tissues). **(C)** Representative IHC staining of PPA1 (brown) in normal ovarian tissues, benign ovarian tumor, borderline ovarian tumor, and EOC tissues. **(D)** PPA1 expression pattern in EOC at different histological stages. **(E)** Representative IHC staining of PPA1 (brown) in EOC with different histological stages. **(F)** Representative IHC staining of PPA1 in EOC at different histological grades. **(G)** Expression of PPA1 in relation to patients’ survival in EOC.

To explore the clinical significance of PPA1 in EOC, the expression of PPA1 was detected in frozen primary EOC samples (n=26) and normal ovarian tissues (n=8) using qRT-PCR analysis (Figure 1A). PPA1 expression was upregulated in EOC tissues compared to PPA1 expression in normal ovarian tissues (*p*<0.05). The protein levels of PPA1 were also measured in frozen EOC tissues (n=3) and normal ovarian tissues (n=3) using Western blots. PPA1 was also upregulated in EOC tissues compared to normal ovarian tissues (Figure 1B).

IHC was performed to examine PPA1 expression in EOC (n=55), borderline tumors (n=20), benign tumors (n=24) and normal ovarian tissues (n=23). We found that PPA1, which was expressed predominantly within cytoplasm, was generally weak or negative in both normal ovarian and benign tumor tissues. In contrast, strong PPA1 expression was observed in both the borderline ovarian tumors and EOC. Compared to normal ovarian tissues and benign tumor tissues, PPA1 was upregulated in EOC and borderline tumors (*p*<0.05). Compared to borderline epithelium tumors, PPA1 was significantly in EOC tissues (*p*<0.05). In short, PPA1 expression was found to be positively correlated with increased tumor malignancy (*p*<0.01) (Table 1, Figure 1C, and Supplementary Figure 1).

**Table 1:** Expression in relation to increased tumor malignancy

	Negative		Weak		Strong		*P* value
	n	Percent	n	Percent	n	Percent	
normal	15	65.2%	8	34.8%	0	0.0%	<0.01
benign*	8	33.3%	15	62.5%	1	4.2%	
borderline*∆	1	5.0%	12	60.0%	7	35.0%	
malignant*∆▲	0	0%	20	36.4%	35	63.6%	

*compare to normal, *P*<0.05; □ compare to benign, *P*<0.05; ▲ compare to borderline, *P*<0.05.

### PPA1 expression and clinical pathological characteristics

The relationship between PPA1 expression and clinical pathological variables was analyzed using the χ^2^ method or Fisher's exact test (Table 2). Patients in stage II (n=7), stage III (n=17), and stage IV (n=7) exhibited stronger PPA1 expression than patients in stage I (n=24) (*p*=0.011; Figure 1D and 1E). When investigating PPA1 expression and histological grades, it was found that PPA1 has the potential to inhibit EOC differentiation (G1=16, G2=13, G3=26; *p*=0.037; Figure 1F), suggesting that PPA1 contributes to the clinical progression of human EOC. There was no correlation observed between PPA1 expression and pathologic type (*p*=0.658, Supplementary Figure 2) or age (*p*=0.098).

**Table 2:** Expression of PPA1 in relation to clinicalpathological parameters

	Weak		Strong		P value
	n	Percent	n	Percent	
Types					0.918
Serous adenocarcinoma	4	28.6%	10	71.4%	
Mucinous adenocarcinoma	5	45.5%	6	54.5%	
Endometrioid carcinoma	5	41.7%	7	58.3%	
Clear cell carcinoma	3	33.3%	6	66.7%	
Low differentiated adenocarcinoma	3	33.3%	6	66.7%	
Histological grade					0.049
1	10	62.5%	6	37.5%	
2	3	23.1%	10	76.9%	
3	7	26.9%	19	73.1%	
Stage					0.011
I	14	58.3%	10	41.7%	
II	1	14.3%	6	85.7%	
III	5	29.4%	12	70.6%	
IV	0	0.00%	7	100%	
Age					0.270
>50	10	45.5%	12	54.5%	
≤50	10	36.4%	23	63.6%	

Follow-up assessments with patients who underwent surgery at the Tianjin Center Hospital of Gynecology Obstetrics and the Tianjin First Center Hospital from 2005 to 2009 were conducted until the end of 2014 in this study. Kaplan-Meier curve analysis indicated that patients with strong PPA1 expression (n=35) tended to have a poor prognosis compared to patients with weak PPA1 expression (n=20; *p*=0.0042; Figure 1G).

### PPA1 in EOC cell line migration *in vitro*

**Figure 2:**
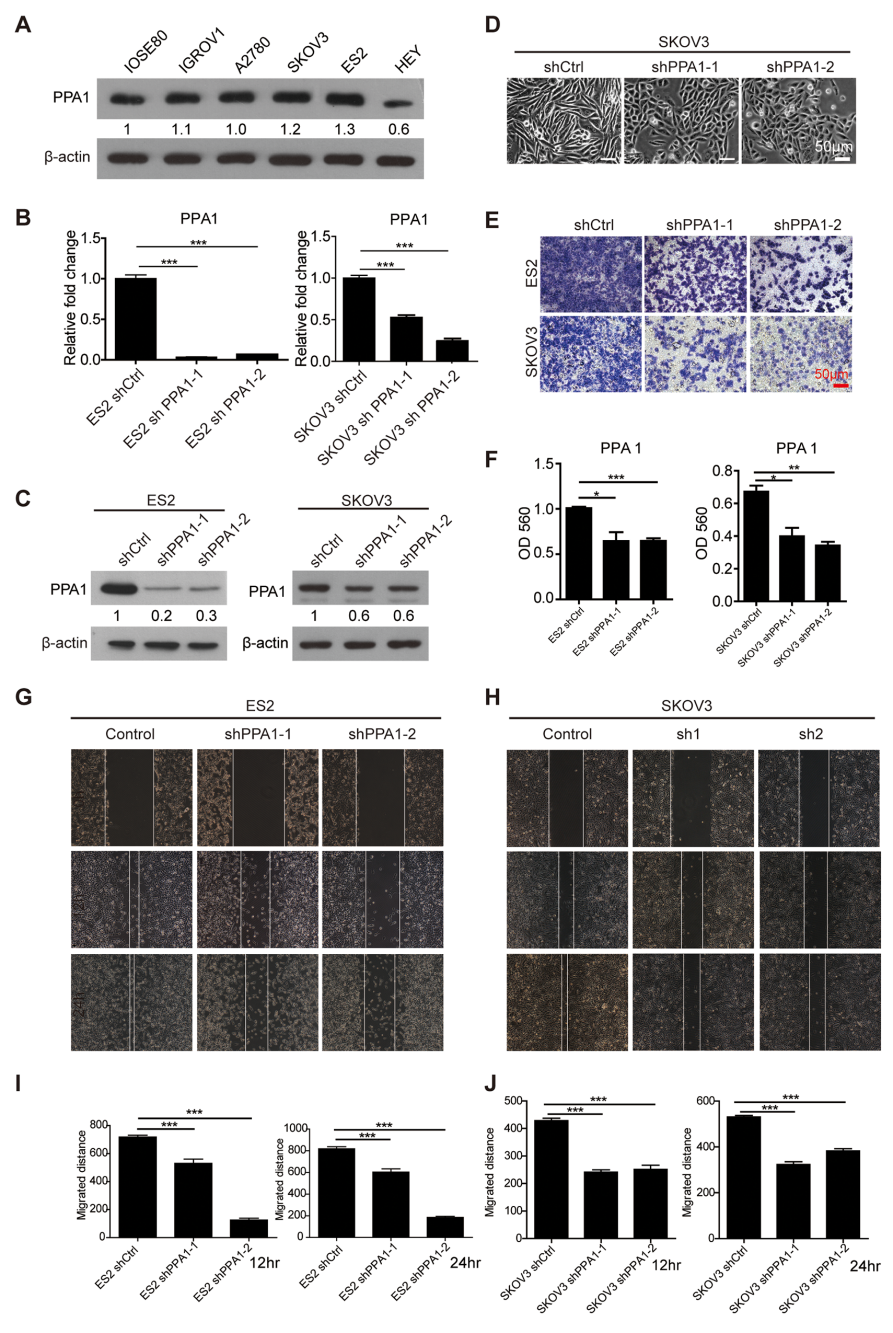
Silencing PPA1 suppresses EOC migration *in vitro* **(A)** The expression of PPA1 in cell lines IOSE80, IGROV1, A2780, SKOV3, ES2, and HEY was detected by western blot. **(B, C)** Silencing PPA1 in ES2 and SKOV3 cells decreased PPA1 expression, which was detected by qRT-PCR and western blot. **(D)** Cell morphology of PPA1 silenced SKOV3 cells and control cells. **(E)** Representative crystal violet staining for ES2 and SKOV3 cells that migrated to the bottom of transwell filters after 12 hours. **(F)** Silencing PPA1 in ES2 and SKOV3 cells decreased migration to the bottom of transwell filters (measured by the absorbance at 560 nm, n=3). **(G, H)** Wound healing images of ES2 and SKOV3 at 0, 12, and 24 hours, respectively. **(I, J)** Statistical analysis of wound healing rates. The wound area relative to the control group is shown as the mean ± SD, n = 3.

According to our clinical observations, PPA1 may promote EOC metastasis. Five EOC cell lines, IGROV1, A2780, SKOV3, ES2, HEY, and the normal ovarian epithelium cell line IOSE80 were chosen for the next functional investigation. In EOC cell lines, the expression of PPA1 was examined with western blots. PPA1 expression in ES2 and SKOV3 was increased slightly compared to IOSE80 (Figure 2A). To further investigate the role of PPA1 in tumor malignancy, 2 shRNAs targeting PPA1 were transfected into the PPA1 high-expression EOC cell lines ES2 and SKOV3 via a lentivirus system. The knockdown efficiency of each shRNA was validated by qRT-PCR (Figure 2B) and western blots (Figure 2C). After PPA1 knockdown, SKOV3 cells lost their mesenchymal-like morphology with a spindle shape, and acquired an epithelial appearance (Figure 2D).

The effects of PPA1 in cell proliferation, migration, and EMT were investigated. We found PPA1 failed to affect cell proliferation (Supplementary Figure 3). The results of the transwell assay (Figure 2E and 2F) and wound healing assay (Figure 2G, 2H, 2I and 2J) suggested that downregulation of PPA1 decreased migration ability compared to control cells for both the ES2 and SKOV3 cell lines.

### Expression of EMT-associated markers after silencing PPA1

**Figure 3:**
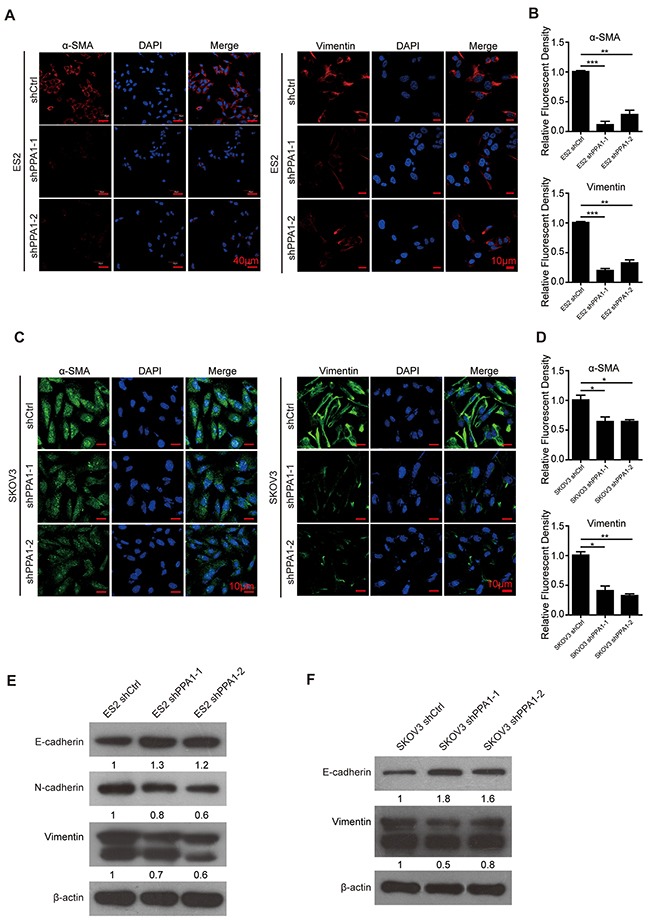
Silencing PPA1 induced reversion of EMT in EOC cells **(A-D)** Immunofluorescence staining of α-SMA and Vimentin in ES2 cells (A) and SKOV3 cells (C). Nuclei were stained with DAPI, and the fluorescence density was quantified. (n = 3; *P < 0.05, **P < 0.01). **(E, F)** Western blotting showed that silencing PPA1 increased E-cadherin expression and decreased N-cadherin and Vimentin expression in ES2 cells and SKOV3 cells.

Using immunofluorescent staining, we detected the levels of EMT-associated proteins α-SMA and Vimentin in the SKOV3 and ES2 cell lines. The mesenchyme-specific markers α-SMA and Vimentin decreased in cells with silenced PPA1 (Figure 3A–3D). Western blot analysis showed that the expression of the epithelium-specific marker E-cadherin was increased, whereas mesenchymal markers N-cadherin and Vimentin were decreased in ES2-shPPA1 cells compared to control cells (Figure 3E). Increased E-cadherin expression and decreased Vimentin expression was also observed in SKOV3 cells. However, N-cadherin expression could not be detected in SKOV3 cell lines (Figure 3F).

**Figure 4:**
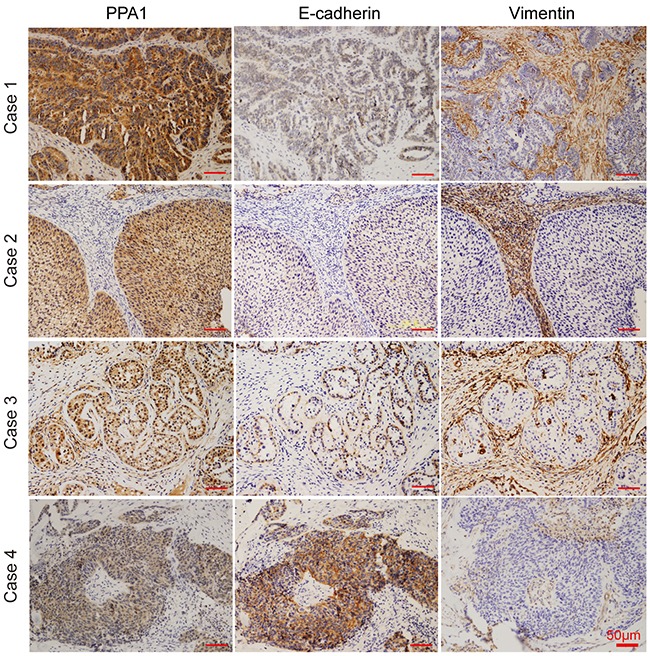
IHC staining revealed correlations between PPA1 and E-cadherin or Vimentin in EOC samples Fifty-five ovarian cancer samples were stained with PPA1, E-cadherin, and Vimentin primary antibodies using serial sections. The upper photographs (Case 1 and 2) represent typical higher PPA1 expression, which displayed lower E-cadherin and higher Vimentin expression profiles. The lower photograph (Case 3 and 4) represent typical cases with lower PPA1 expression, which displayed higher E-cadherin and lower Vimentin expression profiles.

We examined the expression of PPA1, E-cadherin, and Vimentin in 55 human EOC samples. EOC tissues with high PPA1 levels appeared to have high Vimentin expression and low E-cadherin expression. Conversely, EOC tissues with low PPA1 expression exhibited high E-cadherin expression and low Vimentin expression (Figure 4; Table 3). Pearson correlation analysis was employed to investigate the correlation between PPA1 and EMT in EOC. The results indicated that PPA1 expression was negatively associated with E-cadherin expression (R=−0.121, *p*=0.017), and positively associated with Vimentin expression (R=0.362, *p*=0.06). Therefore, PPA1 was closely correlated with decreasing epithelial traits and increasing mesenchymal characteristics.

**Table 3:** Association between PPA1, E-cadherin and Vimentin expression in EOC tissues

Variable expression	Weak(PPA1)		Strong(PPA1)		R	*P* value
	n	percent	n	percent		
E-cadherin					-0.121	0.017
negative	8	42.1%	11	57.9%		
weak	5	19.2%	21	80.8%		
strong	7	70.0%	3	30.0%		
Vimentin					0.362	0.026
negative	7	63.6%	4	36.4%		
weak	7	46.7%	8	53.3%		
strong	6	20.7%	23	79.3%		

### Silencing of PPA1 inhibited EMT by preventing β-catenin to translocate to the nucleus

**Figure 5:**
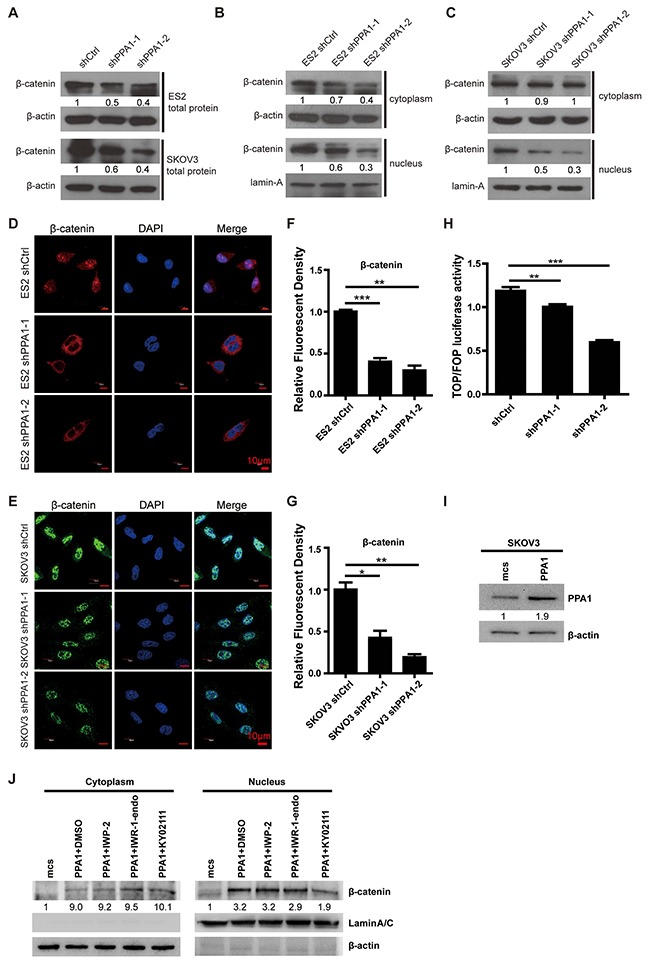
Silencing PPA1 suppresses Wnt/β-catenin signaling pathway **(A)** Silencing PPA1 decreased β-catenin expression from whole cell lysates in ES2 and SKOV3. **(B, C)** Silencing PPA1 down-regulated nuclear β-catenin expression. **(D, E, F, G)** β-catenin translocation was inhibited in PPA1-silenced ES2 and SKOV3 cells compared to control cells, by immunofluorescence. **(H)** TCF activation was measured with a dual-luciferase reporter gene assay, and silencing PPA1 inhibited TCF activation. **(I)** Overexpression of PPA1 in SKOV3 cells was validated by western blotting. **(J)** β-catenin expression in cytoplasm and nucleus of SKOV3-PPA1 cells after treatment with Wnt/β-catenin-specific inhibitors by western blotting.

As previously reported, the Wnt/β-catenin signaling pathway promotes EMT in cancer development [19, 20]. To explore how PPA1 promotes EMT in EOC cells, we performed western blotting and immunofluorescent staining to examine β-catenin expression in total cell, nucleus, and cytoplasm lysates. Western blots showed that total β-catenin expression was decreased in PPA1-silenced cells compared to control cells (Figure 5A). As predicted, nuclear β-catenin expression was down-regulated in PPA1-silenced cells. Cytoplasmic β-catenin levels were not altered in SKOV3 cells, and decreased slightly in ES2 cells with PPA1 knockdown (Figure 5B and 5C). Immunofluorescence staining results showed less nuclear translocation of β-catenin in PPA1 knockdown cells compared to control cells (Figure 5D–5G). TopFlash/FOPFlash reporter genes were used to measure the transactivation of TCF after silencing PPA1. Decreased luciferase activity was detected from the TopFlash reporter for the PPA1-silenced group compared to the scrambled control group (Figure 5H).

To clarify PPA1's effect on the Wnt/β-catenin pathway, we established SKOV3-PPA1 cells which overexpressed PPA1 in the SKOV3 cell line (Figure 5I). The SKOV3-PPA1 cells were treated with a series of Wnt/β-catenin-specific inhibitors. IWP-2 is a Wnt-specific inhibitor that prevents palmitoylation of Wnt protein, therefore blocking Wnt secretion and activity. IWR-1-endo inhibits Wnt-induced accumulation of β-catenin through stabilization of the destruction complex member AXIN2. KY021111 selectively inhibits the Wnt/β-catenin pathway by targeting downstream GSK3β. The GSK3β inhibitor blocked the PPA1-induced β-catenin translocation. We infer that PPA1 may promote β-catenin dephosphorylation (Figure 5J).

### Silencing PPA1-inhibited metastasis of EOC in the syngeneic mouse model

**Figure 6:**
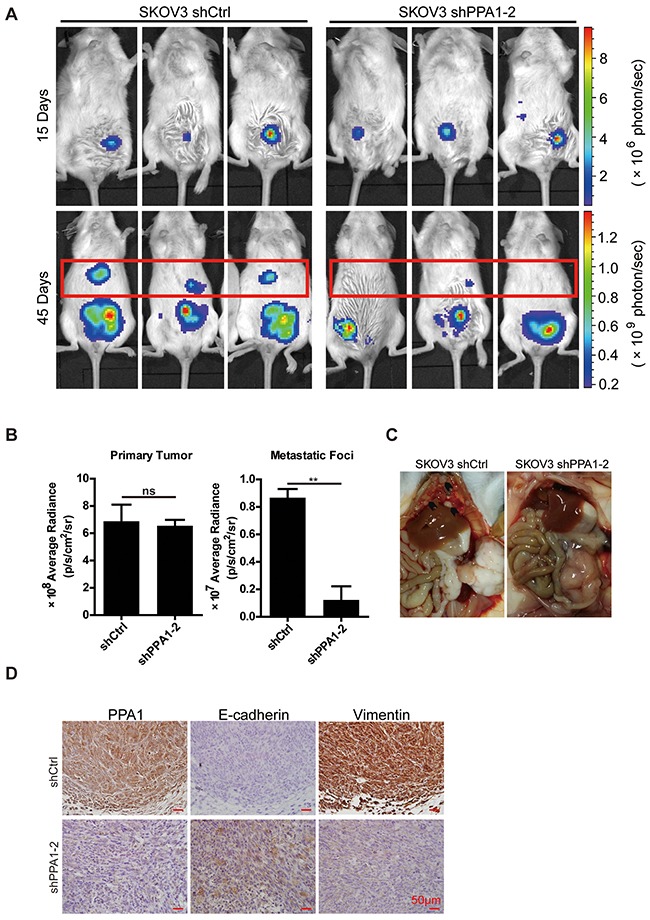
PPA1 in SKOV3 cells accelerating tumor cell metastasis in mouse tumor xenografts *in vivo* **(A)** SKOV3 shPPA1-2 and shCtrl cells were injected intraperitoneally into NOD/SCID mice, and the bioluminescence images of xenografted tumors were taken at the times indicated. **(B)** Bioluminescence intensity was measured and plotted, n = 4. **(C)** Anatomic images showing metastases in the liver surface and sub-mesentery in xenograft tumors of shCtrl cells, whereas xenograft tumors in the shPPA1-2 group were detected in the injection site. **(D)** The representative IHC staining of PPA1, E-cadherin, and Vimentin in the xenografted tumor tissues of NOD/SCID mice.

To validate the correlation between PPA1 expression and tumor cell metastasis, a xenografted tumor model was performed. NOD/SCID mice were intraperitoneally (i.p.) injected with SKOV3 shCtrl (n=4) or SKOV3 shPPA1-2 cells (n=4). Forty-five days after injection, primary and metastatic tumors were measured based on the luminescence of luciferase. Photon counts decreased in the metastatic site of shPPA1-2, but did not change in primary tumors (Figure 6A and 6B). Mice injected with shPPA1-2 cells showed less ascetic fluid accumulation in the abdominopelvic cavity compared to control animals (data not shown). Three of the mice injected with SKOV3 shCtrl cells developed metastases (multiple small metastatic nodules) under the diaphragm and liver surface, whereas none of the mice injected with the shPPA1-2 cells exhibited metastatic tumors (Figure 6C). The results indicate that silencing PPA1 can inhibit metastasis of SKOV3 cells *in vivo*.

We investigated the expression of PPA1, E-cadherin, and Vimentin in xenografted mouse EOC tumor slices. As predicted, the shCtrl group with high PPA1 expression had high levels of Vimentin and low levels of E-cadherin. Conversely, xenografted tumor tissues from the shPPA1-2 group expressing low levels of PPA1 exhibited a strong E-cadherin and weak Vimentin expression profile (Figure 6D).

## DISCUSSION

As a pyrophosphatase driving biosynthetic reactions, PPA1 was found to be upregulated in multiple cancers. From our previous study [18], we identified PPA1 as a biomarker candidate through proteomic analyses, which was confirmed by using tissue microarrays. The present study was designed to evaluate PPA1 expression in different types of human ovarian tumor tissues and normal ovarian tissues to investigate how PPA1 functions.

Based on several results, high PPA1 expression can promote the occurrence and development of ovarian cancer, and PPA1 expression is positively correlated to a poor prognosis for ovarian cancer. First, compared to normal ovarian tissues and benign ovarian tumors, we found that PPA1 was overexpressed in human EOC tissues. Clinical pathological analysis showed PPA1 expression is positively correlated with increases in FIGO stage and histological grade. Second, higher PPA1 expression in EOC tissues decreased the five-year survival rate. Third, depletion of PPA1 suppressed migration of ES2 and SKOV3 EOC cells. Fourth, tumor metastasis was inhibited after PPA1 was silenced *in vivo*.

Metastasis in ovarian cancer is established by the EMT-driven delamination of ovarian cancer cells from the primary tumor *in situ,* and subsequent penetration into the surrounding peritoneal cavity [21–23]. Based on our results, we showed that PPA1 may promote EMT. First, the EOC cell line SKOV3 gained epithelial characteristics and lost mesenchymal-like properties after PPA1 was silenced. Second, western blot and immunofluorescence revealed that silencing of PPA1 down-regulated the expression of markers associated with mesenchymal cells (e.g., N-cadherin, α-SMA, or Vimentin), while it up-regulated the expression of epithelial cell markers (e.g., E-cadherin). Third, PPA1 expression was negatively associated with E-cadherin expression, but positively correlated with Vimentin expression.

In our study, nuclear β-catenin expression decreased after PPA1 was silenced compared to cells in the control group. We demonstrated that the regulatory effect of PPA1 in EMT in EOC cells might promote the activation of the Wnt/β-catenin signaling pathway and the β-catenin nuclear translocation.

In summary, our study demonstrated that PPA1 was overexpressed in EOC with advanced grades and stages, and high PPA1 expression correlated with poor survival of EOC patients. PPA1 expression increased metastasis by stimulating EMT both *in vitro* and *in vivo*. Furthermore, the Wnt/β-catenin signaling pathway promoted this process via translocation of β-catenin. Thus, our data support PPA1 as a potential marker for evaluating EOC progression and a useful target for EOC therapy.

## MATERIALS AND METHODS

### Clinical samples and cell lines

Formalin-fixed, paraffin-embedded human specimens, including EOC (n=55), borderline tumors (n=20), benign tumors (n=24), and normal ovarian tissues (n=23), were collected from patients who underwent surgery at the Tianjin Central Hospital of Gynecology Obstetrics and the Tianjin First Center Hospital between 2005 and 2009 (n=122). The classification of clinical staging and histologic grading of EOC were determined according to the FIGO 2006 system. Patient ages ranged from 25 to 67. Within the follow-up period of 60 months, 24 patients died of the disease. Among these 122 patients, 34 fresh tissue samples (EOC=26, normal ovarian tissues=8) were snap frozen and stored at -80°C to obtain RNA and protein samples. Normal ovary specimens were sectioned from patients with uterine fibroids during total abdominal hysterectomy and bilateral salpingo-oophorectomy and were diagnosed by post-operative pathological examination. Approval from the Institutional Research Board at these two hospitals was obtained prior to the study.

Human EOC cell lines (IGROV1, A2780, SKOV3, ES2, HEY) and a human ovarian surface epithelial cell line (IOSE80) were stored at the Nankai University School of Medicine. The cell lines were purchased from ATCC and cultured according to the instructions provided. Human embryonic kidney 293T (HEK293T) cells were cultured in Dulbecco's Modified Eagle Medium (Corning, USA) enriched with 10% FBS, 1% P/S, 2% L-glutamine, 1% Sodium pyruvate and 1% non-essential amino acid. All cells were grown at 37°C and 5% CO2 in a humidified incubator.

### Immunohistochemistry

Immunohistochemical (IHC) staining was carried out as previously described [24]. Five micrometer sections of paraffin-embedded human EOCs, borderline tumors, benign tumors, and normal ovarian tissues were prepared for staining. After dewaxing, the sections were rehydrated, then antigen retrieval and endogenous peroxidase blocking were performed. The slides were incubated with monoclonal antibodies (PPA1: Sigma, St. Louis MO, 1:250; E-cadherin: BD Bioscience, San Jose CA, 1:200; Vimentin: Cell signaling Technology, Danvers, MA, 1:200) overnight at 4°C. The sections were then incubated with biotinylated anti-mouse (PPA1, E-cadherin) or anti-rabbit (Vimentin, β-catenin) antibody (vector laboratories, Burlingame, CA) followed by HRP-streptavidin. Antigens were detected with peroxidase substrate and counterstained with hematoxylin. Scoring was based on the intensity and proportion of positively stained cells. The intensity of positive staining was scored from 0 to 3 as follows: 0 (none), 1 (low), 2 (moderate), and 3 (high). The proportion was scored from 0 to 4 as follows: <10% was scored as 0; 10–25% was scored as 1; 26–50% was scored as 2; 51–75% was scored as 3 and >75% was scored as 4. These two values were multiplied to produce the total score. A score <2 was considered PPA1-negative, 2–5 was considered PPA1 weak staining, and 6-12 was considered PPA1 strong staining.

### Quantitative reverse transcription-polymerase chain reaction (qRT-PCR)

Total RNA was extracted from cells or tissues and reverse-transcribed to cDNA. The qRT-PCR procedure was performed (BioRad CFX96 realtime PCR machine, CA) with TransStart Green qPCR SuperMix (Transgene, China). The specific primers for human GAPDH were 5’-CTCTGATTTGGTCGTATTGGG-3’ (sense primer) and 5’-TGGAAGATGGTGATGGGATT-3’ (anti-sense primer). The specific primers for human PPA1 were 5’-CGCTATGTTGCGAATTTGTTC-3’ (sense primer) and 5’-CCAGTATGTTTATCATTGTGCC-3’ (anti-sense primer). After denaturation at 94°C for 30 s, amplification was performed in 40 cycles at 94°C for 5 s, 53°C for 15 s, and 72°C for 10 s.

### Western blotting

For protein expression analysis, cells or tissues were lysed, proteins were separated with 10% SDS-polyacrylamide gel electrophoresis, then transferred onto polyvinylidene difluoride membranes (Millipore). Immunoblots were performed with primary monoclonal antibodies for PPA1 (Sigma, St. Louis MO), β-actin (Santa Cruz, Dallas, Texas), E-cadherin (BD Bioscience, San Jose CA), N-cadherin (BD Bioscience, San Jose CA), Vimentin (Cell signaling Technology, Danvers, MA), β-catenin (Abcam, Cambridge, UK), and Lamin-A (Cell signaling Technology, Danvers, MA). Next, the blots were incubated with horseradish peroxidase-linked goat-anti-mouse (PPA1, β-actin, E-cadherin, N-cadherin) or goat-anti-rabbit (Vimentin, β-catenin, Lamin-A) secondary antibody. The signals were detected with the Chemilucent ECL Detection system (Millipore). The results of the western blots were analyzed using Image J software. The values were listed below the bands.

### Plasmid and lentivirus-based gene transduction

The shRNA sequences targeting PPA1 (PPA1-shRNA-1: 5’AAAAGCTACTGTGGACTGGTTTATTGGATCCAATAAACCAGTCCACAGTAGC-3’, PPA1-shRNA-2: 5’-AAAAGGAATCAGTTGCATGAATATTGGATCCAATATTCATGCAACTGATTCC-3’ and a control sequence (shCtrl: 5’-GCAGTTATCTGGAAGATCAGGTTGGATCCAACCTGATCTTCCAGATAACTGC-3’) were synthesized and inserted into a pLV-H1-EF1α-Puro vector (Biosettia, San Diego, CA). Recombinant pLV-H1-EF1α-Puro lentiviruses were packaged in 293T cells following the manufacturer's protocol (Biosettia, San Diego, CA) and transduced into ES2 and SKOV3 cells as previously described [25]. Transduced cells were purified with 1.0 μg/ml puromycin (Sigma-Aldrich).

### Immunofluorescence cell staining

Cells were incubated with α-SMA (BD Bioscience, San Jose CA, 1:200), Vimentin (Cell signaling Technology, Danvers, MA, 1:200), and β-catenin (Rabbit, Abcam, Cambridge, UK, 1:200) overnight at 4°C. The cells were then stained with Alexa Fluor^@^ 594- or Alexa Fluor^@^488- labeled secondary antibodies (Abbkine, 1:1000) at room temperature for 1 hour, and counterstained with DAPI for 5 minutes. Images were acquired using confocal microscopy under a 40× objective (FV1000-IX81, Olympus Microsystems, Shanghai, China).

### Transwell and wound healing assays

Cells from each sample group were seeded into the top chamber of a 24-well plate with 4 μm polyethylene terephthalate membrane inserts (Millipore). The chamber was filled with RPMI-1640 (Corning, USA) containing 10% FBS, and the inserts were filled with 1% FBS RPMI-1640. After 12h incubation, the filter membrane was fixed with 100% methanol and stained with crystal violent for another 12h, then washed with 50% acetic acid. The OD value was measured by microplate reader (Promega). Cell migration was determined by the mean ± SD.

For the wound healing assay, ES2 and SKOV3 cells were plated for 24 hours in a 6-well plate. A scratch was made in the confluent cell layer using a sterile tip. Medium was changed and replaced with RPMI 1640 with 2% FBS. Cellular migration was assessed after 12 h and 24 h.

### Luciferase reporter gene assay

A TOPFlash reporter plasmid containing two sets of three copies of the transfection grade T-cell factor (TCF) binding site upstream of the Luciferase open reading frame was used in the assay. A FOPflash plasmid containing mutated TCF binding sites was included as a negative control. TOPFlash and FOPFlash reporter plasmids (Millipore, CA) were individually co-transfected with shRNA (shPPA1-2 and shCtrl) and a plasmid with a Renilla reporter gene into 293T cells. After 48 h, cells were lysed with lysis buffer, and the activity of each reporter genes activity was detected with a Dual-Luciferase® Reporter Assay (Promega, Madison, WI).

### Tumor xenograft and bioluminescence imaging

Female NOD/SCID mice, 6-8 weeks old, were kept under Specific Pathogen Free conditions according to Beijing Medical Experimental Animal Care guidelines. The animal experiments were approved by the Institutional Animal Care and Use Committee of Tianjin Nankai University School of Medicine. Ovarian cancer cells (SKOV3) stably transfected with firefly luciferase and shRNA (shPPA1-2 and shCtrl) were generated, and 1.25×10^6^ cells were injected intraperitoneally into the NOD/SCID mice. Bioluminescence imaging to determine the fate of transplanted cells in living mice was performed as previously described [26]. Briefly, mice underwent BLI to determine firefly luciferase expression using the *in vivo* imaging system (IVIS 200) (Xenogen Corporation, Hopkinto, MA). Following anesthesia with 2% isoflurane, mice were injected with D-Luciferin IP (150 mg/kg; Biosynth International, Naperville, IL) and 1 s to 5 min scans were performed to assess firefly luciferase expression.

### Statistical analysis

All of the data were presented as mean ± standard deviation. Statistical analyses were conducted using SPSS 17.0 software. A chi-square (χ^2^ test) test, Pearson's correlation test and Student's t-test were used. A *p* < 0.05 was used as the criterion for statistical significance.

## SUPPLEMENTARY MATERIALS FIGURES AND TABLES


