# Supplementary material for: Silencing PPA1 inhibits human epithelial ovarian cancer metastasis by suppressing the Wnt/β-catenin signaling pathway
Source: Oncotarget. 2017 Jul 18;8(44):76266–78. doi: 10.18632/oncotarget.19346 (PMC5652704; doi:10.18632/oncotarget.19346)
Supplement: Supplementary file 1 [file oncotarget-08-76266-s001.pdf]

Downregulation of HADH promotes gastric cancer progression via Akt signaling pathway

SUPPLEMENTARY MATERIALS

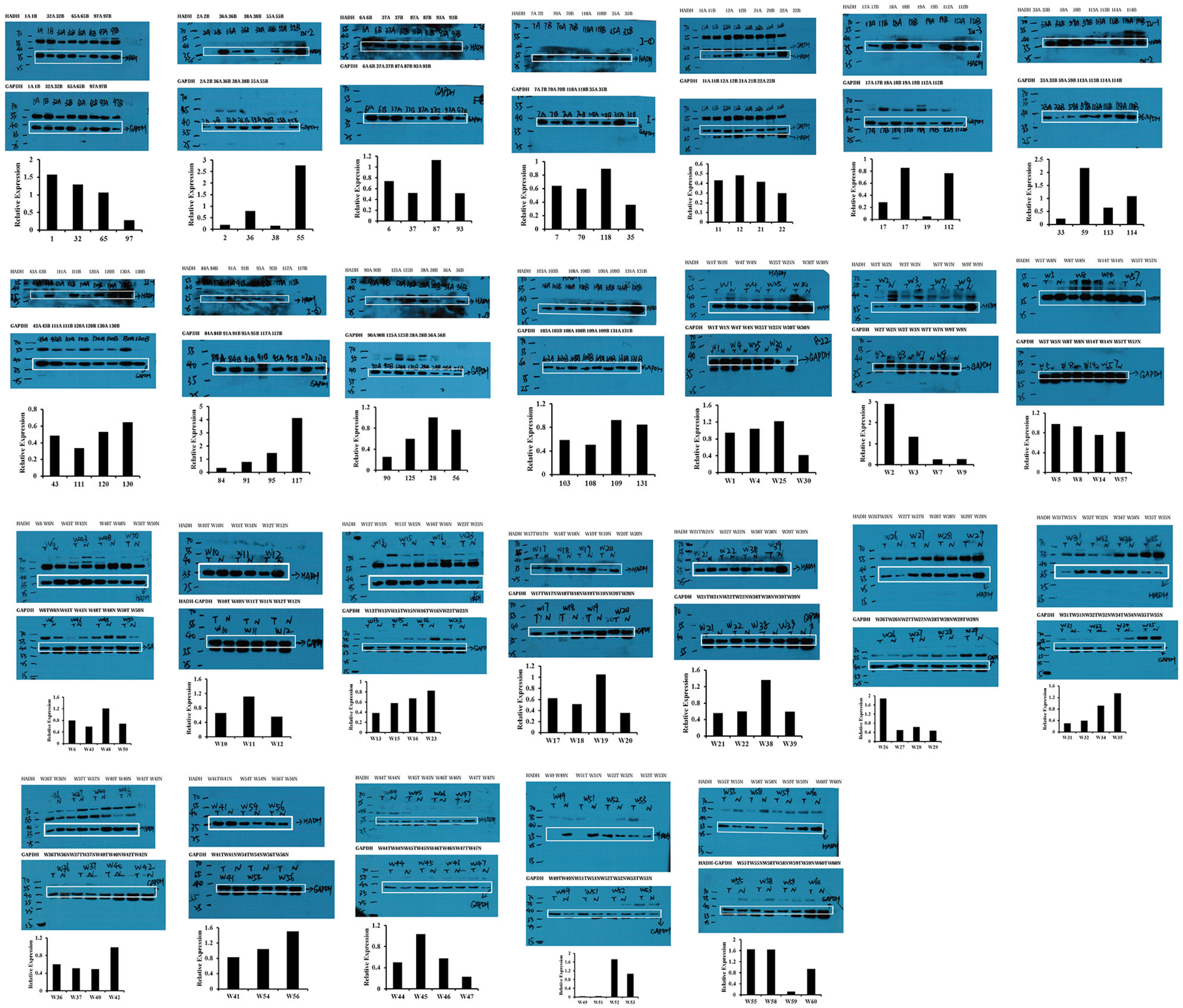

Supplementary Figure 1: Uncropped images of HADH expression in gastric cancer samples. HADH expression was analyzed in 102 pairs of GC samples and matched adjacent normal gastric tissues by Western Blot. (**A** or WT: cancer, **B** or WN: adjacent normal gastric tissue).

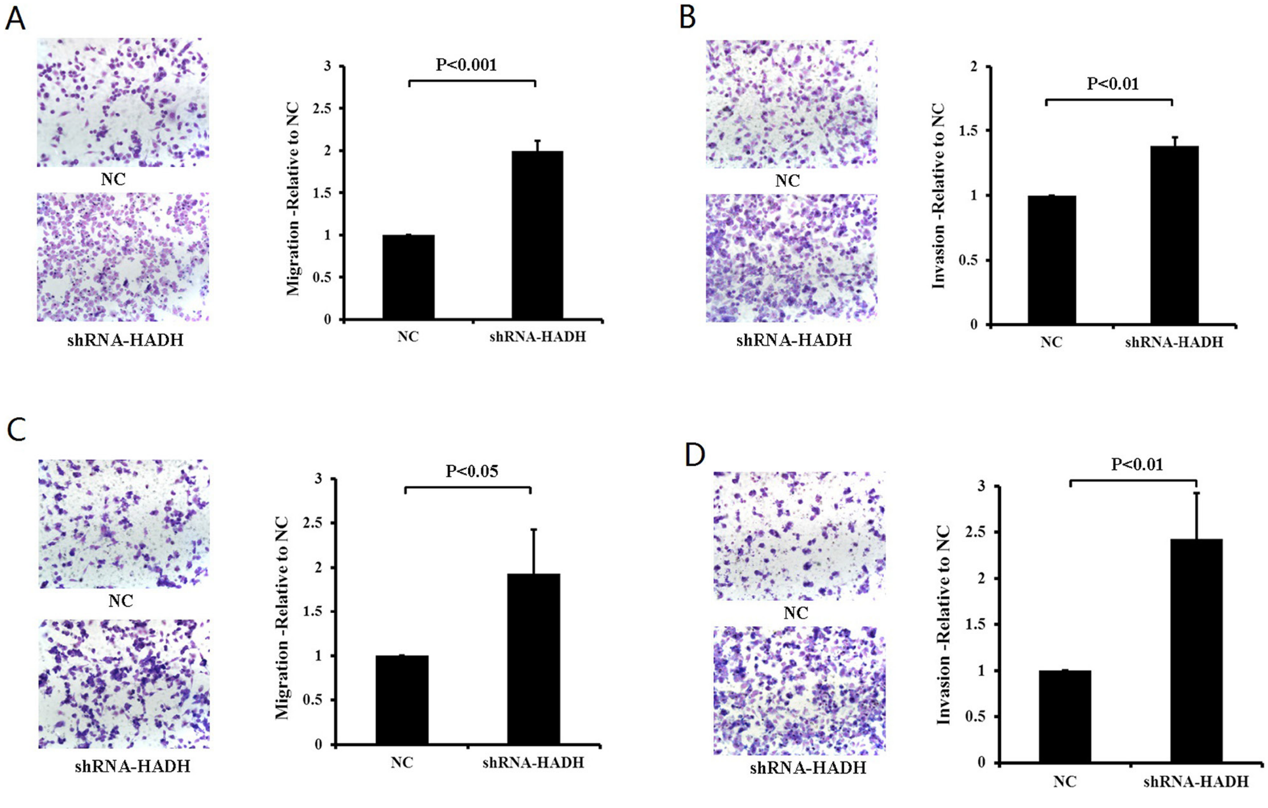

Supplementary Figure 2: Knockdown of HADH by shRNA promotes AGS and N87 cell migration and invasion. Migration and invasion assays were conducted in HADH shRNA- or NC-transfected cells by using 24-well Transwell chambers. Cell migration and invasion was assessed by counting the number of cells that migrated or invaded through the transwell insert in 3 independent membranes by using light microscopy, then normalized against the NC-treated cells to determine the relative ratio. **(A)** Representative images of migrated AGS cells transfected with NC or shRNA. **(B)** Representative images of invaded AGS cells transfected with NC or shRNA. **(C)** Representative images of migrated N87 cells transfected with NC or shRNA. **(D)** Representative images of invaded N87 cells transfected with NC or shRNA.

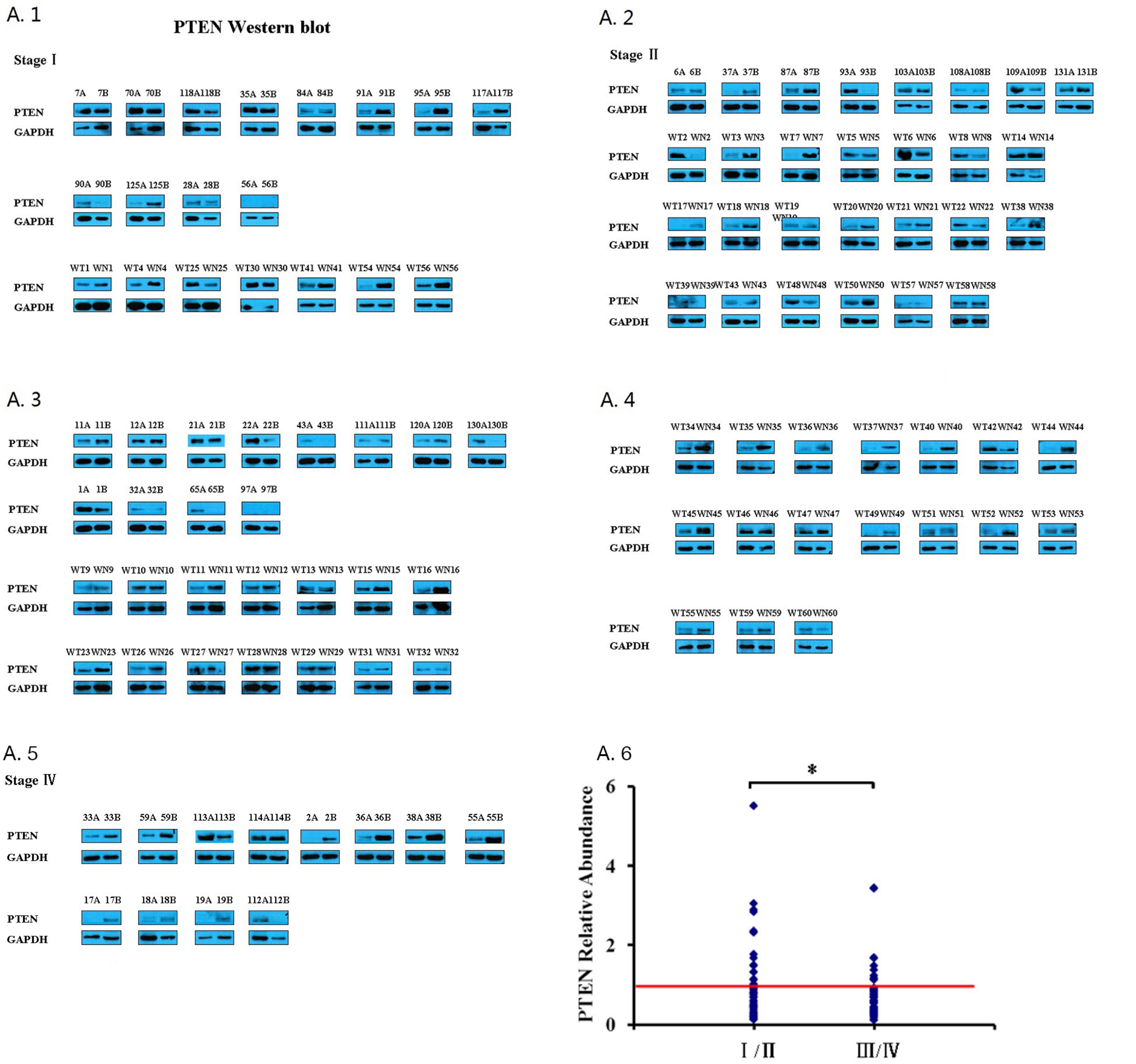

Supplementary Figure 3: Western blot and real-time PCR analysis of PTEN expression in gastric cancer samples. (A) PTEN expression was analyzed in 102 pairs of GC samples and matched adjacent normal gastric tissues by Western Blot. ***(continued)***

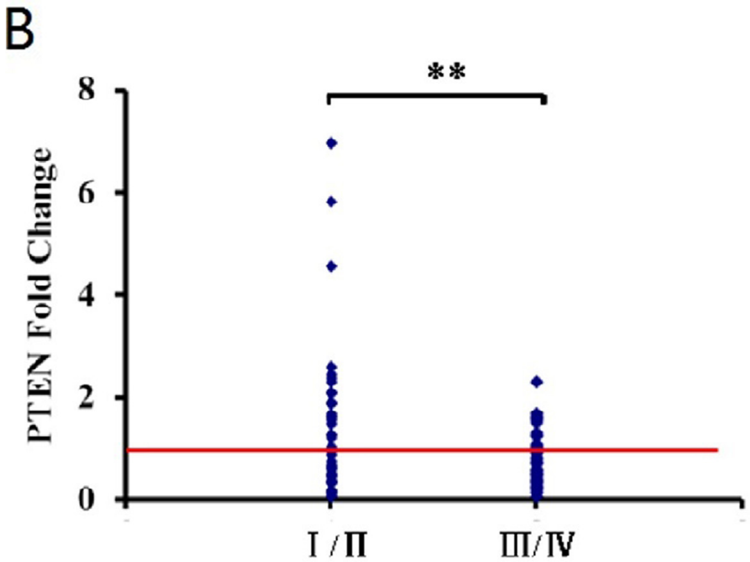

Supplementary Figure 3: *(Continued)* Western blot and real-time PCR analysis of PTEN expression in gastric cancer samples. (B) PTEN mRNA levels were analyzed in 102 pairs of GC samples and matched adjacent normal gastric tissues by real-time PCR. (A or WT: cancer, B or WN: adjacent normal gastric tissue).
